# National survey of mentorship in Canadian general surgery residency programs: Where are we and what do we need?

**Published:** 2017-12-15

**Authors:** Megan Delisle, Justin Rivard, Pamela Hebbard, Brendan McCarthy, Debrah Wirtzfeld

**Affiliations:** 1 Section of General Surgery, Faculty of Health Sciences, University of Manitoba, Manitoba, Canada; 2 Center for Healthcare Innovation, University of Manitoba, Manitoba, Canada

## Abstract

**Background:**

The benefits of mentorship on residents are well established. The current state of mentorship in General Surgery (GS) residency programs in Canada is unknown. The objectives of this study were to obtain GS residents’ and program directors’ (PD) perspectives on resident mentorship.

**Study Design:**

An electronic survey was developed and distributed to all 601 GS residents in Canada. All 17 PDs were invited for telephone interviews.

**Results:**

A total of 179 of the 601 residents responded. Ninety-seven percent (n=173) felt mentorship was important. Only 67% (n=116) identified a mentor and only 53% (n=62) reported a mentorship program. Most who identified a mentor (n=87/110, 79%) were satisfied with the mentorship received. Significant variations in mentorship existed between demographic subgroups and mentorship program types. Overall, residents (n=121, 74%) favoured having a required mentorship program.

A total of 11 out of 17 PDs participated in the telephone interviews. The majority of PDs (n=9, 82%) were satisfied with current resident mentorship but most acknowledged that barriers exist (n=8, 73%).

**Conclusion:**

GS programs in Canada should ensure they are providing equal opportunities for mentorship across demographic subgroups. Programs are encouraged to examine both their program’s and their residents’ needs as well as local barriers to improve mentorship.

## Introduction

Over the last decade, the number of applicants to surgical residencies has declined and one out of five surgical residents do not complete their training.^1^,^2^ The burnout rate among Canadian General Surgery residents was found to be 34%, higher than most other specialties and the general population.^2^–^4^ A link between career satisfaction, resident retention and attrition and mentorship has been recognized.^2^,^3^,^5^–^8^ Mentorship is associated with increased promotions, successful research grants and publications.^9^–^19^ Residents can seek guidance and help from their mentors for difficult cases, operative skills, employment opportunities and networking.^11^–^14^,^16^ Currently, there are no standards for or data on mentorship among General Surgery residency programs in Canada or the United States. Mentorship is critical to the formation of residents’ professional identity and sense of belonging and to implement effective mentorship solutions, more information is needed.^20^–^27^

Our hypothesis is there is a lack of emphasis and resources placed on the development and maintenance of mentorship relationships among General Surgery residents in Canada. Thus, the objectives of our study were to obtain Canadian General Surgery residents’ and program directors’ critical perspectives on current resident mentorship with the goal of implementing or strengthening mentorship programs across the country.

## Methods

Ethics approval was obtained from the University of Manitoba Research Ethics Board.

### Definition

We adopted the most common definition of mentorship in the surgical literature, consistent with vertical mentorship: “the process whereby a more experienced, usually senior, individual (the mentor) guides the personal and professional development of someone more junior (the mentee).”^14^,^28^–^30^ We defined informal mentorship programs as those not requiring documentation and/or a certain number of encounters.^28^–^30^ Formal mentorship programs, on the other hand, were defined as those requiring documentation and/or a certain number of encounters.^28^–^30^

### Study design

An e-mail invitation requesting participation in an online, anonymous survey on mentorship was sent to all General Surgery residents in Canada (n=601). The first invitation was sent in September 2015 followed by a two-week reminder. The survey was open for one month. Two $50 Starbucks gift cards were raffled to increase participation. An invitation to participate was included in the September and October 2015 Canadian Association of General Surgeons monthly newsletter.

An e-mail invitation requesting participation in a semi-structured telephone interview was sent to all General Surgery program directors (n=17) in April 2016 with one two-week reminder. No response after the two-week reminder was interpreted as a decline to participate. Questions were not provided to program directors prior to the telephone interview. Telephone interviews were conducted by the primary investigator (M.D.) and were standardized using three questions. Interviews were recorded and transcribed. A semi-structured telephone interview was chosen to improve participation and to obtain richer data than possible with a survey.

### Survey

An electronic survey (Supplementary Material) was created using previous literature.^9^,^11^,^28^–^30^ The questionnaire was reviewed by five experts in the field of mentorship and piloted by over 20 surgical residents outside of General Surgery with a range of seniority and specialty to establish content validity. The survey was administered using a professional online interface, namely SurveyMonkey.com^®^. Test-retest and inter-observer reliability were deemed unnecessary.^31^,^32^ The survey utilized a real-time modification of the questions offered depending on the respondents’ answers and the total number of questions could range from 5 to 25. Guidelines on questionnaire research and an expert in survey development from the Center for Healthcare Innovation at the University of Manitoba were consulted throughout survey development.^31^

### Mentorship effectiveness scale

The Mentorship Effectiveness Scale was included in the survey.^32^ This 12-question scale was originally developed by an Ad Hoc Faculty Mentoring Committee at Johns Hopkins University. It was intended to objectively measure mentorship effectiveness among healthcare students. Content validity was obtained by the developing committee in the faculty of nursing. We adapted the Mentorship Effectiveness Scale to make it more relevant. We updated the wording and then combined two questions we felt were redundant. There were 11 questions in the end, each with 6 points ranging from 0 to 5 (Supplementary Material). The minimum possible score was 0 and the maximum was 55, with higher scores representing higher effectiveness. Written permission to use and modify the scale was obtained from Dr. Ronald Berk.

### Statistical analysis

Program size was collected as a categorical variable based on number of residents (0–10, 11–20, 21–30, 31–40, 41–50, 51–60 and ≥61) and was dichotomized using the median. Resident training level was collected as a continuous variable and dichotomized using commonly accepted definitions of a junior resident (≤2 years of training) and a senior resident (≥ 3 years of training).

Statistical data analysis was performed using SPSS (IBM Corp. Released 2012. IBM SPSS Statistics for Windows, Version 21.0. Armonk, NY: IBM Corp). Dichotomous outcomes and dichotomous/categorical predictors were compared using chi-square analyses. A factorial ANOVA with 95% confidence intervals (CI) and standard error of the means (SEM) were calculated to determine if mentorship program type influenced residents’ Mentorship Effectiveness Scale scores. Chi-square analyses were performed to determine if: 1) resident satisfaction was associated with type of mentorship program or how mentor was obtained and 2) resident preference of mentorship program type was associated with year of training or residency program size. A two-sided p-value equal to or less than 0.05 was considered significant.

## Results

### Survey results

#### Demographics

A total of 179 General Surgery residents (30%) completed the questionnaire (Table 1).

#### Prevalence of reported mentorship

Ninety-seven percent (n=173) of General Surgery residents in Canada who answered the questionnaire felt that mentorship was important to their training, but only 67% (n=116) could identify a mentor. Significant differences in reported prevalence of mentorship among demographic subgroups existed between males and females (Table 1).

#### Reasons for obtaining a mentor

Of the 116 residents who identified a mentor, 115 responded to the question regarding how they obtained their mentor. Of these, 79% (n=91) obtained a mentor on their own. Men more commonly obtained a mentor because the mentor was approachable or easy to work with compared to women (n=40, 93% vs. n=36, 75%, respectively; p=0.02) (Table 2). Residents from larger programs more commonly reported obtaining a mentor because they were in their sub-specialty of interest compared to residents from smaller programs (n=24, 52% vs. n=14, 31%, respectively; p=0.04) (Table 3).

#### Reasons for not obtaining a mentor

Significant differences in reasons for not obtaining a mentor existed among juniors and seniors (Table 4). Compared with seniors, more juniors reported not obtaining a mentor because they were unfamiliar with potential mentors (n=5, 19% vs. n=0, respectively; p=0.01). Compared with juniors, more seniors reported not obtaining a mentor because mentorship was not supported by their residency program (n=13, 43% vs. n=3, 11%, respectively; p=0.01).

#### Mentorship characteristics

Sixty-seven percent (n=76) of the 115 residents who identified a mentor had more than one mentor. Mentors were most frequently obtained in residents’ junior years (n=86, 74%) compared to their senior years (n=15, 13%) and clerkship (n=6, 5%). Mentors included an attending (n=100, 86%), a more senior resident (n=30, 26%), the program director (n=14, 12%) or a fellow (n=7, 6%).

#### Status of mentorship programs

Of the residents who identified a mentor, 53% (n=62) stated that a formal or informal mentorship program existed in their residency program (Table 5). Significant differences in resources for mentorship and frequency of communication with mentors existed between formal, informal and no programs (Table 5).

#### Outcomes of mentoring

Seventy-nine percent (n=87) of residents reported being satisfied with their mentors and 21% (n=23) felt neutral. Resident satisfaction with mentoring was not significantly associated with type of mentorship program (p=0.51) or if the mentor was assigned versus obtained by the resident (p=0.7).

Significant differences in mentorship satisfaction existed between demographic subgroups. Ninety-five percent of residents with mentorship programs (n=56) felt their mentors were accessible compared to only 83% with no program (n=39; p=0.04). Junior residents were less satisfied with mentors’ guidance on professional and personal issues (n=29, 76% and n=19, 50%, respectively) compared to senior residents (n=65, 90%; p=0.05 and n=52, 72%, respectively; p=0.02).

#### Mentor effectiveness

Average Mentorship Effectiveness Scale scores ranged from 46 (SEM ±1.5, 95% CI 43–49) for informal programs compared to 48 (SEM ±1.9, 95% CI 44–52) for formal programs, with 55 representing the highest attainable score. The average score for mentors that were assigned was similar to scores if mentors were self-obtained (47 [SEM ±1.7, 95% CI 43–50]) versus 47 [SEM ±1.2, 95% CI 45–50], respectively). Mentorship Effectiveness Scale scores were not significantly associated with type of mentorship program or how residents obtained their mentors (p=0.6 and p=0.89, respectively). The observed power for measuring Mentorship Effectiveness Scale scores by type of mentorship program was β=0.13. The observed power for measuring Mentorship Effectiveness Scale score by how mentor was obtained was p=0.05.

#### Difficulties in mentorship

The most common difficulties identified with mentor-mentee relationships included time constraints (n=65, 59%) and scarcity of qualified mentors (n=29, 26%). Residents with no mentorship programs felt that scarcity of qualified mentors (n=20, 48%) was more of a difficulty compared to residents with a mentorship program (n=9, 15%; p= <0.01).

#### Ideal mentorship

Residents felt that the most important attributes of an ideal mentor include:

- Someone who works in General Surgery or a General Surgery subspecialty (n=152, 93%)
- Someone who is chosen by them (n=89, 54%)
- Someone who does not have an influence on their academic standing (n=68, 41%).

Gender (n=23, 14%), age (n=16, 10%), and ethnicity/religion (n=3, 2%) were less important attributes. However, 19% of women (n=18) valued having a mentor of the same gender compared to only 7% of men (n=5; p=0.03).

Significant differences in desired subjects for mentorship existed between demographic subgroups. Women reported wanting mentorship on professional development (n=85, 97%) and clinical confidence (n=71, 81%) significantly more commonly than men (n=56, 82%; <p=0.01 and n=43, 63%; p=0.16) (Table 2C). Residents from larger programs reported wanting mentorship on personal life significantly more frequently than residents from smaller programs (n=44, 55% vs. n=27, 36%; p= 0.01) (Table 3C). Finally, juniors reported wanting mentorship on operative skills significantly more frequently than seniors (n=51, 82% vs. n=64, 68%; p=0.05) (Table 4C).

Residents from larger programs favored an interest based matching process compared to smaller programs (n=61, 73% vs. n=42, 52% respectively; p=0.01). Men (n=24, 35%) more commonly favored having topics to stimulate discussion with their mentors compared to women (n=14, 15%; <p=0.01).

The majority of residents (n=121, 74%) felt that a mentorship program should be required. Mentorship program preference did not vary significantly with year of residency training (p=0.5) or residency program size (p=0.63).

#### Program director interviews

Eleven program directors (65%) completed the telephone interview.

##### Question 1: How do residents obtain mentors?

Only one program reported having a formal mentorship program. Four reported assigning mentors only to residents needing remediation. The remaining six programs had no mentorship program and these program directors reported being primarily responsible for facilitating resident mentorship. These same program directors felt that they were the primary mentor for most of their residents.

##### Question 2: Are residents satisfied with the current mentorship situation in your program?

Eighty-two percent of program directors (n=9) felt that residents were satisfied with the current mentorship situation. One program reported having a formal mentorship program in the past but discontinued it due to lack of success with assigned mentors. In particular, this program director felt mentorship was more successful if self-initiated and was contemplating starting an informal mentorship program where mentors were self-selected. The program directors felt that some residents were “falling through the cracks” since the elimination of the program. Three program directors contemplated starting a formal mentorship program, but felt that mentorship is most meaningful when it is organic.

##### Question 3: What are the current problems with mentorship in your program?

Two program directors stated that it was possible some residents were not finding mentors due to lack of a mentorship program. Another stated that it was challenging because of generational gaps and professionalism issues. Two felt overwhelmed by having to take on the role of a mentor for all their residents. One program director felt that the main challenge with mentorship is time constraints. Two program directors felt that there was a lack of resources, including dedicated time off, faculty development and financial compensation.

## Discussion

Our study found that only two-thirds of respondents currently identify a mentor, despite almost all understanding its importance. The prevalence of mentorship in Canadian General Surgery programs appears to be higher than that reported in other studies (28% – 65%).^11^–^14^,^19^,^28^,^29^,^33^ The results of our survey highlight some interesting differences in mentorship among demographic subgroups.

The first comparison is between men and women. Women more often reported not having a mentor compared to men. A mentorship gap among women in medicine has been previously identified.^30^,^34^ Similar to other studies, our study showed a same gender mentor was more important for women.^10^,^35^ This may limit the possible available mentors for female residents and can put a strain on currently practicing female surgeons as the number of female surgical residents has increased dramatically in the last decade.^36^ Lack of mentorship has commonly been cited as a cause for women wanting to leave their current job position and for their lack of career advancement.^10^ A recently published prospective cohort study by Yeo et al. found that being female was the independent factor most strongly associated with attrition from a surgical residency program.^37^ It is imperative that residency programs begin to address this disparity.

The second interesting comparison is between junior and senior residents. Our survey showed junior residents more commonly reported not obtaining a mentor because they were unfamiliar with potential mentors compared to senior residents. Mentorship is most critical during junior years as these are when burnout and attrition rates are the highest.^2^,^37^ This has been postulated to be due to lack of mechanisms to discuss personal and professional concerns in a safe, non-judgmental environment.^2^,^37^ These issues are as important as the traditional reasons people seek mentors (i.e., for career planning). Junior residents can be overwhelmed by the transition to residency and may not have the courage to approach mentors in their new environment. Furthermore, they may not yet understand the importance and relevance of mentorship thus not making it a priority. We hypothesize that junior residents would benefit more from a structured, formal mentorship program compared to senior residents.^15^ It is critical to provide junior residents with a framework to help take ownership of and become an active mentee, a concept known as “managing up” in the business world.^38^,^39^ Providing mentors and mentees with appropriate training to develop and nurture respectful mentorship relationships early in residency could help level the playing field for junior residents.^23^

Another important comparison can be made between formal, informal and no mentorship programs. The importance of structured mentorship programs is highlighted in our data. Residency programs with formal and informal programs had significantly more resources for mentorship and had more active mentorship relationships compared to those with no program. Only 53% of respondents in our study reported a formal or informal mentorship program.^23^ Previous research has shown the prevalence of mentorship rises to 77 – 90% when mentorship programs are in place.^17^,^40^ In the interviews, program directors appeared reluctant to accept mentorship programs, as they believed it negates the ability to form organic mentorship. The debate between organic versus assigned mentors is long standing and research exists highlighting the benefits of both.^40^–^43^ Our results demonstrated that there was not a significant difference in resident satisfaction or mentorship effectiveness based on program type or if a mentor was assigned versus self-selected. In addition, residents favored having a mentorship program. This can serve as evidence that mentorship programs and assigned mentors are not detrimental to mentorship relationships and may actually be preferred by residents. Contemporary residency training, characterized by short clinical rotations on busy services with rapid turnover, may not be as conducive to organic mentorship. Thus, mentorship programs may be a way to enhance organic mentorship by providing residents and faculty the resources necessary to develop and sustain future mentorship.

A reassuring finding in our study is that General Surgery residents’ satisfaction with current mentorship is considerably higher than all other Canadian residency programs (79% vs. 13%, respectively).^27^ This means that efforts to improve mentorship need to be well targeted not to disturb the benefits of what is already occurring. Residents’ level of training, demographics and program size need to be taken into account as their mentorship needs vary greatly as demonstrated by our results. Residents need to take an active role in establishing appropriate mentorship for themselves and requesting the resources they need to develop their own roles as mentors. Furthermore, our results demonstrate that the burden of mentorship appears to fall on surgeons with only a small portion of residents identifying seniors and fellows as mentors. One potential strategy to increase mentor participation could be to provide resources for surgeons, fellows, and senior residents to understand and develop their role in mentoring more junior residents and medical students.^23^ Kashiwagi et al. found seven types of mentoring models utilized in medicine: dyad, peer, facilitated peer, speed, functional, group, and distance mentoring.^44^ The most effective strategy depends on the individual and may even be a combination of types to create what is known in the literature as a mosaic of mentors, “*a team of mentors who work well together and have complimentary skill sets.*”^45^

### Limitations

Our data were obtained via self-reporting and are therefore subject to bias. Residents who did not respond may be systematically different than those who responded. For example, the most common respondents may have been those who were particularly satisfied or unsatisfied with their mentor, a phenomenon known as the social desirability bias.^46^ Furthermore, responses may have been subject to the halo or horn effect, where an overall impression of a mentor overshadowed their individual traits.^32^

A response rate of 30% represents a favorable number for an online, voluntary, anonymous survey, but it may not be representative of all 601 General Surgery residents in Canada. Although our response rate is low, it is on par with or higher than similar studies conducted among residents in North America.^11^–^13^,^47^ The timing of our study may have influenced the results as first year residents were beginning their residency and may have identified mentors later in the year. The low observed power was limited by the size of the convenience sample. This did not allow for extensive statistical comparison. Even with these limitations, the data presented are novel and important for the development of mentorship for General Surgery residents.

### Conclusion

There are variations in mentorship among General Surgery residents in Canada. Efforts need to be made to improve available resources while respecting that each resident’s mentorship requirements are unique. One possible strategy may involve standardizing mentorship through accreditation to help level the disparities that exist among residents. Stakeholders need to understand that investing the resources in mentorship today is important for training the mentors of tomorrow and will strengthen the postgraduate medical education system.^24^,^25^

## Supplementary Information



## Figures and Tables

**Table 1:** Distribution of demographics by presence of mentor

	Mentor n=116 (%)	No Mentor n=57 (%)	P-value

**Sex**			**0.04**

Female	60 (52)	39 (68)	
Male	56 (48)	18 (32)	

**Age (y)**			0.46

21–25	12 (10)	7 (12)	
26–30	61 (53)	35 (61)	
31–35	32 (28)	14 (25)	
26–40	9 (8)	1 (2)	
>/=40	2 (2)	0	

**Level in residency training**			0.1

Junior (≤ PGY-2)	40 (34)	27 (47)	
Senior (≥ PGY-3)	76 (66)	30 (53)	

**Size of training program**			0.39

Small (≤30 residents)	55 (47)	31 (54)	
Large (≥ 31 residents)	61 (53)	26 (46)	

**Table 2:** Variations in mentorship in women versus men

	Women n (%*)	Men n (%*)	P-value

**A. Reasons for obtaining a mentor**	**48 (100)**	**43 (100)**	

Mentor was studying area of interest in research	18 (38)	20 (47)	0.38
Mentor fulfilled need for a research adviser	21 (44)	18 (42)	0.85
Mentor was easy to work with/approachable	36 (75)	40 (93)	0.02
Person had a good reputation as a mentor	8 (17)	14 (33)	0.08
Mentor was in sub-specialty I was interested in	18 (38)	20 (47)	0.38
Mentor had a practice environment that I saw as ideal	12 (25)	10 (23)	0.85
I wanted to get a job	3 (6)	5 (12)	0.37

**B. Reasons for not obtaining a mentor**	**39 (100)**	**18 (100)**	

Time constraints	15 (39)	5 (28)	0.43
Generational gap	2 (5)	1 (6)	0.93
Personality conflicts	3 (8)	1 (6)	0.77
Opposite gender available, prefer same gender	1 (3)	0	0.49
Same gender available, prefer opposite gender	0	0	
Scarcity of qualified mentors	7 (18)	4 (22)	0.7
Cannot identify someone who truly reflects what you need	21 (54)	7 (39)	0.30
Do not want someone who is also an educational supervisor	7 (18)	1 (6)	0.21
Unfamiliar with potential mentors	4 (10)	1 (6)	0.56
Not supported by residency program	10 (26)	6 (33)	0.55

**C. Desired topics of mentorship**	**88 (100)**	**68 (100)**	

Professional development	85 (97)	56 (82)	<0.01
Career decisions	85 (97)	63 (93)	0.26
Academic/research	63 (72)	46 (68)	0.59
Exam performance	44 (50)	33 (49)	0.85
Operative skills	65 (74)	50 (74)	0.96
Clinical confidence	71 (81)	43 (63)	0.01
Personal life	43 (49)	28 (41)	0.34

* Total may be greater than 100% as residents could select more than one answer.

**Table 3:** Variations in mentorship in small (≤30 residents) versus large (≥31 residents) residency programs

	Small n (%*)	Large n (%*)	P-value

**A. Reasons for obtaining a mentor**	**45 (100)**	**46 (100)**	

Mentor was studying area of interest in research	18 (40)	20 (44)	0.74
Mentor fulfilled need for a research adviser	21 (47)	18 (39)	0.46
Mentor was easy to work with/approachable	36 (80)	40 (87)	0.37
Person had a good reputation as a mentor	7 (16)	15 (33)	0.06
Mentor was in sub-specialty I was interested in	14 (31)	24 (52)	0.04
Mentor had a practice environment that I saw as ideal	9 (20)	13 (28)	0.36
I wanted to get a job	4 (9)	4 (9)	0.97

**B. Reasons for not obtaining a mentor**	**31 (100)**	**26 (100)**	

Time constraints	12 (39)	8 (31)	0.53
Generational gap	1 (3)	2 (8)	0.45
Personality conflicts	1 (3)	3 (12)	0.22
Opposite gender available, prefer same gender	1 (3)	0	0.36
Same gender available, prefer opposite gender	0	0	
Scarcity of qualified mentors	5 (16)	6 (23)	0.5
Cannot identify someone who truly reflects what you need	16 (52)	12 (46)	0.68
Do not want someone who is also an educational supervisor	6 (19)	2 (8)	0.21
Unfamiliar with potential mentors	4 (13)	1 (4)	0.23
Not supported by residency program	7 (23)	9 (35)	0.32

**C. Desired topics of mentorship**	**76 (100)**	**80 (100)**	

Professional development	68 (90)	73 (91)	0.7
Career decisions	71 (93)	76 (95)	0.67
Academic/research	54 (71)	54 (68)	0.63
Exam performance	37 (49)	39 (49)	0.99
Operative skills	58 (76)	56 (70)	0.38
Clinical confidence	56 (74)	57 (71)	0.74
Personal life	27 (36)	44 (55)	0.01

* Total may be greater than 100% as residents could select more than one answer.

**Table 4:** Variations in mentorship in juniors (≤ 2 years of training) versus seniors (≥3 years of training)

	Juniors n (%*)	Seniors n (%*)	P-value

**A. Reasons for obtaining a mentor**	**26 (100)**	**65 (100)**	

Mentor was studying area of interest in research	11 (42)	27 (42)	0.94
Mentor fulfilled need for a research adviser	10 (39)	29 (45)	0.6
Mentor was easy to work with/approachable	22 (85)	54 (83)	0.86
Person had a good reputation as a mentor	6 (23)	16 (25)	0.88
Mentor was in sub-specialty I was interested in	8 (31)	30 (46)	0.18
Mentor had a practice environment that I saw as ideal	6 (23)	16 (25)	0.88
I wanted to get a job	1 (4)	7 (11)	0.29

**B. Reasons for not obtaining a mentor**	**27 (100)**	**30(100)**	

Time constraints	11 (41)	9 (30)	0.4
Generational gap	1 (4)	2 (7)	0.61
Personality conflicts	1 (4)	3 (10)	0.35
Opposite gender available, prefer same gender	1 (4)	0	0.29
Same gender available, prefer opposite gender	0	0	
Scarcity of qualified mentors	5 (19)	6 (20)	0.89
Cannot identify someone who truly reflects what you need	13 (48)	15 (50)	0.89
Do not want someone who is also an educational supervisor	4 (15)	4 (13)	0.87
Unfamiliar with potential mentors	5 (19)	0	0.01
Not supported by residency program	3 (11)	13 (43)	0.01

**C. Desired topics of mentorship**	**62 (100)**	**94 (100)**	

Professional development	54 (87)	87 (93)	0.25
Career decisions	58 (94)	90 (96)	0.54
Academic/research	42 (68)	67 (71)	0.63
Exam performance	34 (55)	43 (46)	0.27
Operative skills	51 (82)	64 (68)	0.05
Clinical confidence	49 (79)	65 (69)	0.17
Personal life	26 (42)	45 (48)	0.46

* Total may be greater than 100% as residents could select more than one answer.

**Table 5:** Variations in mentorship between program types^+^

Resource	Formal program n=16 (%*)	Informal program n=46 (%*)	No program n=53 (%*)	P-value
Protected time for meetings	10 (63)	6 (13)	0	<0.02
Merit for outstanding mentor	3 (19)	15 (33)	5 (10)	0.02
Objectives for mentors and/or mentees (i.e., suggested meeting frequency)	14 (88)	6 (13)	5 (10)	<0.01
Case discussions and readings to stimulate discussion	6 (38)	11 (24)	1 (2)	<0.01
List of available mentors	3 (19)	10 (22)	1 (2)	0.01
Interest based matching process	1 (6)	10 (22)	0	<0.01
Not aware of any	0	18 (39)	47 (89)	<0.01
**Frequency of communication**	**Formal program** n=16 (%*)	**Informal program** n=46 (%*)	**No program** n=51 (%*)	
Minimum once a month	2 (13)	19 (41)	10 (20)	0.02
Minimum every six months	7 (44)	3 (7)	4 (8)	<0.01
Ad-hoc	7 (44)	23 (50)	36 (71)	0.05
**Duration of relationship**	**Formal program** **n=15 (%)**	**Informal program** **n=44 (%)**	**No program** **n=48 (%)**	
<1 year	6 (40)	16 (36)	11 (23)	0.27
1–3 years	6 (40)	25 (57)	27 (56)	0.49
>3 years	3 (20)	3 (7)	10 (21)	0.14

Informal mentorship programs were defined as those not requiring documentation and/or a certain number of encounters. Formal mentorship programs were defined as those requiring documentation and/or a certain number of encounters.^28^–^30^

* Total may be greater than 100% as residents could select more than one answer.
