# Supplementary material for: National survey of mentorship in Canadian general surgery residency programs: Where are we and what do we need?
Source: Can Med Educ J. 2017 Dec 15;8(4):e42–53. (PMC5766218)
Supplement: Supplementary file 1 [file CMEJ-08-42-s001.pdf]

Thank you for your participation.

Enter your email address below for a chance to winone of two 50$ Starbucks gift cards. Otherwise, your email address, personal information and IP address will not be recorded. All answers will remain anonymous and confidential. There are no risks to participating in this survey and the University of Manitoba Health Research Ethics Board has approved the study.

If you have any questions about this study, please contact us at delislem@myumanitoba.ca.

By continuing on and completing the survey you are consenting to participate.

1. Enter email address (optional)

About you

* 2. Are you male or female?

- ◯ Female
- ◯ Male

* 3. What is your age (years)?

- ◯ </= 20
- ◯ 21-25
- ◯ 26-30
- ◯ 31-35
- ◯ 36-40
- ◯ >40

* 4. What year of residency training are you in?

- ◯ 1
- ◯ 2
- ◯ 3
- ◯ 4
- ◯ 5
- ◯ 6
- ◯ >/=7

* 5. Approximately how many residents are in your program?

- ◯ Do not know
- ◯ 0-10
- ◯ 11-20
- ◯ 21-30
- ◯ 31-40
- ◯ 41-50
- ◯ 51-60
- ◯ >/=61

Mentoring in Surgery

What do we mean by mentoring?

The process whereby a more experienced, usually senior, individual (the mentor) guides the personal and professional development of someone more junior (the mentee).

* 6. What is your perspective on mentors in surgery?

- ◯ Critical to my training
- ◯ Beneficial, but not critical to my training
- ◯ Not important to my training
- ◯ Obstructive to my training
- ◯ Neutral/no opinion

Mentoring in Surgery

* 7. How many mentors do you have?

- ◯ 0
- ◯ 1
- ◯ 2
- ◯ >/=3

Mentoring in Surgery

* 8. Which of the following best describes mentorship in your program?

- ◯ A formal mentorship program exists (i.e. formal documentation of encounters)
- ◯ An informal mentorship program exists (i.e. no formal or required documentation)
- ◯ No program exists
- ◯ Unsure

* 9. Mark all resources available for mentorship in your residency program:

- ☐ Protected time for meetings
- ☐ Merit for outstanding mentors
- ☐ Objectives for mentors and/or mentees (i.e. suggested meeting frequency)
- ☐ Case discussions and readings to stimulate discussion
- ☐ List of available mentors
- ☐ Interest based matching process
- ☐ Not aware of any
- ☐ Other (please specify)

* 10. How often are you required to communicate with your mentor?

- Weekly
- Monthly
- Each rotation
- Every six months
- Yearly
- Ad-hoc/ no requirement
- Other (please specify)

Mentoring in Surgery

* 11. How often do you communicate with your mentor?

- Weekly
- Monthly
- Each rotation
- Every six months
- Yearly
- Ad-hoc
- Other (please specify)

Mentoring in Surgery

* 12. How did you obtain your mentor?

- ○ Assigned by institution, program director, etc.
- ○ Obtained on my own
- ○ Was approached by mentor
- ○ Recommended by a colleague

* 13. Which of the following best describes your mentor(s)? (mark all that apply)

- ☐ More senior resident than yourself
- ☐ Fellow
- ☐ Attending
- ☐ Program director
- ☐ Other (please specify)

* 14. What year of residency was your mentor assigned?

- ◯ 1
- ◯ 2
- ◯ 3
- ◯ 4
- ◯ 5
- ◯ Other, please indicate year #:

Mentoring in Surgery

* 15. What year of residency did your mentor approach you?

- ( ) 1
- ( ) 2
- ( ) 3
- ( ) 4
- ( ) 5
- ( ) Other (please indicate year #)

[ ]

Mentoring in Surgery

* 16. What year of residency did you obtain a mentor of your own?

- ◯ 1
- ◯ 2
- ◯ 3
- ◯ 4
- ◯ 5
- ◯ Other (please indicate year #)

* 17. Why did you obtain a mentor on your own (mark all that apply):

- ☐ Mentor was studying area of interest in research
- ☐ Mentor fulfilled my need for a research adviser
- ☐ Mentor was easy to work with/approachable
- ☐ Person had a good reputation as a mentor
- ☐ Mentor was in sub-specialty I was interested in
- ☐ Mentor had a practice environment that I saw as ideal
- ☐ I wanted to get a job
- ☐ Other (please specify)

Mentoring in Surgery

* 18. What reason(s) was your mentor recommended to you (mark all that apply):

- [ ] Mentor was studying area of interest in research
- [ ] Mentor fulfilled my need for a research adviser
- [ ] Mentor was easy to work with/approachable
- [ ] Person had a good reputation as a mentor
- [ ] Mentor was in sub-specialty I was interested in
- [ ] Mentor had a practice environment that I saw as ideal
- [ ] I wanted to get a job
- [ ] Other (please specify)

Mentoring in Surgery

* 19. Through what format does your mentoring most commonly take place?

- ◯ Telephone
- ◯ Email
- ◯ Face-to-face
- ◯ Audio/teleconferance (e.g. Skype, FaceTime)
- ◯ Text message/imessage/instant message
- ◯ Other (please specify)

* 20. Are you aware of your mentor having received training in mentorship/leadership/communcation?

- ◯ Yes
- ◯ No
- ◯ Do not know

* 21. How long has this mentoring relationship existed?

- ◯ 0-5 months
- ◯ 6-11 months
- ◯ 1-3 years
- ◯ 4-5 years
- ◯ >6 years

* 22. Please identify the reason(s) for not having a mentorship relationship (mark all that apply):

- [ ] Time constraints
- [ ] Generational gap
- [ ] Personality conflicts
- [ ] Opposite gender available, prefer same gender
- [ ] Same gender available, prefer opposite gender
- [ ] Scarcity of qualified mentors
- [ ] Cannot identify someone who truly reflects what you need
- [ ] Do not want someone who is also an educational supervisor
- [ ] Other (please specify)

Mentoring in Surgery

* 23. Which of the following best describes mentorship in your residency program?

- ○ A formal mentorship program exists (i.e. formal documentation of encounters)
- ○ An informal mentorship program exists (i.e. no formal or required documentation)
- ○ No program exists
- ○ Unsure

* 24. What resources are available for mentorship in your program? (mark all that apply)

- ☐ Protected time for meetings
- ☐ Merit for outstanding mentors
- ☐ Objectives for mentors and/or mentees (i.e. suggested meeting frequency)
- ☐ Case discussions and readings to stimulate discussion
- ☐ List of available mentors
- ☐ Interest based matching process
- ☐ Not aware of any
- ☐ Other (please specify)

Outcomes of mentoring

* 25. Indicate which of the following you agree/disagree with:

My mentor was...

	strongly disagree	disagree	neutral	agree	strongly agree	not applicable
Accessible	○	○	○	○	○	○
Professional	○	○	○	○	○	○
A content expert	○	○	○	○	○	○
Approachable	○	○	○	○	○	○
Supportive	○	○	○	○	○	○
Motivating	○	○	○	○	○	○

Other (please specify)

Outcomes of Mentoring

* 26. Indicate which of the following you agree/disagree with:

My mentor provided...

	strongly disagree	disagree	neutral	agree	strongly agree	not applicable
Constructive critiques	◯	◯	◯	◯	◯	◯
Guidance on professional issues	◯	◯	◯	◯	◯	◯
Satisfactory answers and resources	◯	◯	◯	◯	◯	◯
Acknowledgement of your contributions	◯	◯	◯	◯	◯	◯
Guidance on personal issues	◯	◯	◯	◯	◯	◯

Other (please specify)

Outcomes of Mentoring

* 27. Overall, how satisfied you are with your mentorship relationship:

- ○ Not satisfied
- ○ Neutral
- ○ Satisfied

* 28. What are the problem(s) with your current mentor-mentee relationship? (mark all that apply)

- ☐ Time constraints
- ☐ Generational gap
- ☐ Personality conflict
- ☐ Opposite gender, prefer same gender
- ☐ Same gender, prefer opposite gender
- ☐ Scarcity of qualified mentors
- ☐ Assigned someone who does not truly reflect what you need
- ☐ Assigned someone who is also an educational supervisor
- ☐ None
- ☐ Other (please specify)

Ideal Mentorship

* 29. Select all attributes of an ideal mentor that are important to you: (mark all that apply)

- [ ] Works in General Surgery or a General Surgery sub-specialty
- [ ] Same ethnicity and/or religion
- [ ] Same gender
- [ ] Similar generation as you
- [ ] Is a clinical academic
- [ ] Is also your educational supervisor
- [ ] Someone who is chosen by you
- [ ] Someone who is allocated to you
- [ ] Does not have an influence on your academic standing
- [ ] Other (please specify)

* 30. You would like a mentor to help with the following aspect(s): (mark all that apply)

- [ ] Professional development
- [ ] Career decisions
- [ ] Academic/research
- [ ] Exam performance
- [ ] Operative skills
- [ ] Clinical confidence
- [ ] Personal life
- [ ] Other (please specify)

Ideal Mentorship

* 31. Select resource(s) that would facilitate the development of a mentorship relationship? (mark all that apply)

- [ ] Protected time for meetings
- [ ] Merit for outstanding mentors
- [ ] Objective for mentors and/or mentees (i.e. suggested meeting frequency)
- [ ] Case discussion and readings to stimulate discussions
- [ ] List of available mentors
- [ ] Interest based matching process
- [ ] Other (please specify)

* 32. What should a mentoring program be?

- ○ Required and monitored by residency program
- ○ Required but not monitored by residency program
- ○ Not required by residency program
- ○ Other (please specify)
